# Substitution impacts of Nordic wood-based multi-story building types: influence of the decarbonization of the energy sector and increased recycling of construction materials

**DOI:** 10.1186/s13021-022-00205-x

**Published:** 2022-05-17

**Authors:** Tanja Myllyviita, Elias Hurmekoski, Janni Kunttu

**Affiliations:** 1 Finnish Environment Institute, Helsinki, Finland; 2 University of Helsinki, Helsinki, Finland; 3 European Forest Institute, Joensuu, Finland

**Keywords:** Climate change, Construction, Greenhouse gas emissions, Substitution, Wood-frame multi-story building

## Abstract

**Background:**

The building and construction sectors represent a major source of greenhouse gas (GHG) emissions. Replacing concrete and steel with wood is one potential strategy to decrease emissions. On product level, the difference in fossil emissions per functional unit can be quantified with displacement factors (DFs), i.e., the amount of fossil emission reduction achieved per unit of wood use when replacing a functionally equivalent product. We developed DFs for substitution cases representative of typical wood-frame and non-wood frame multi-story buildings in the Nordic countries, considering the expected decarbonization of the energy sector and increased recycling of construction products.

**Results:**

Most of the DFs were positive, implying lower fossil emissions, if wood construction is favored. However, variation in the DFs was substantial and negative DFs implying higher emissions were also detected. All DFs showed a decreasing trend, i.e., the GHG mitigation potential of wood construction significantly decreases under future decarbonization and increased recycling assumptions. If only the decarbonization of the energy sector was considered, the decrease was less dramatic compared to the isolated impact of the recycling of construction materials. The mitigation potential of wood construction appears to be the most sensitive to the GHG emissions of concrete, whereas the emissions of steel seem less influential, and the emissions of wood have only minor influence.

**Conclusions:**

The emission reduction due to the decarbonization of the energy sector and the recycling of construction materials is a favorable outcome but one that reduces the relative environmental benefit of wood construction, which ought to be considered in forest-based mitigation strategies. Broadening the system boundary is required to assess the overall substitution impacts of increased use of wood in construction, including biogenic carbon stock changes in forest ecosystems and in wood products over time, as well as price-mediated market responses.

**Supplementary Information:**

The online version contains supplementary material available at 10.1186/s13021-022-00205-x.

## Introduction

The building and construction sectors represent a major source of greenhouse gas emissions (GHGs) [19]. Thus, these sectors play a fundamental role in achieving a low-emissions society. The World Green Building Council [36] estimates that buildings and infrastructure around the world can reach 40% less embodied carbon emissions by 2030 and achieve net zero emissions in new buildings by 2050.

In the past, studies on the climate change impacts of buildings have focused on the use stage of buildings, such as the GHG emissions related to heating and cooling. As the operational GHG emissions of buildings decline because of the decarbonization of the energy sector and energy-efficient construction techniques, more attention is being given to the so-called embodied GHG emissions, i.e., emissions arising from the manufacturing and processing of raw materials [4, 26]. According to a review by Röck et al. [26], the “average share of embodied GHG emissions from buildings following current energy performance regulations is approximately 20–25% of life cycle GHG emissions, this figure escalates to 45–50% for highly energy-efficient buildings and surpasses 90% in extreme cases”. The production of construction materials for new buildings and refurbishment represents 11% of global overall energy and process-related GHG emissions, with more than half of these caused by the manufacturing of steel and cement [20]. Thus, the building sector and the embodied emissions of construction raw materials are two key areas where emission reductions are required.

Replacing emission-intensive raw materials with wood in the construction sector is considered a potential option to decrease embodied GHG emissions [34]. Wood construction is expected to face positive market prospects, which are at least partly driven by claims of its superior environmental performance (e.g., [21, 32]). Wood construction has a long tradition in countries with substantial forest resources and forest industries, such as the Nordic countries. However, new opportunities are also emerging in large-scale construction markets elsewhere, due to recent innovations and growing interest among decision-makers and industries (FAO 38; Hildebrandt et al. 39).

The climate change mitigation impacts of wood use in the construction sector have been extensively assessed in the scientific literature (e.g., [15, 27]). Displacement factors (DFs) are used to describe the efficiency of using wood to reduce fossil GHG emission by quantifying the amount of fossil emission reduction achieved per unit of wood use in a certain application [27]. For instance, a DF of 2 tCO_2_eq./t implies that the use of one ton of wood products in a specific end use to replace a functionally equivalent product leads to an emission reduction of 2 tons of fossil CO_2_eq./yr (not including biogenic emissions, i.e., carbon stored in forests or wood products). DF should capture all fossil GHG emissions into the atmosphere of the compared functional units, including emissions from raw material extraction, processing, transportation, manufacturing, distribution, use, re-use, maintenance, recycling, and final disposal (e.g., [13]. In the case that not all processing stages are considered, the system boundaries need to be clearly defined. According to meta-analyses (e.g. [22, 27], it appears that, on average, wood construction emits less fossil GHG emissions than construction based on concrete and steel, but the anticipated impact is highly influenced by methodological choices and is likely to vary in time.

The substitution impacts of wood products typically focus on the differences in embodied fossil carbon only, while biogenic carbon flows ought to be considered separately as forest carbon stock changes in the LULUCF (Land Use, Land Use Change and Forestry) sector, as agreed in international GHG reporting conventions [24]. That is, wood energy is reported as zero emissions at the point of combustion to avoid double-counting, because the equivalent emission is captured in the biogenic carbon stock changes when harvesting wood. Thus, the sink impact of changes in forest carbon stocks and in the wood product pool are typically not considered when comparing the climate change mitigation impact of wood construction with alternative construction methods on a product level. However, in a broader context of climate change mitigation, the emissions avoided through wood use should compensate for carbon loss in forests in order to decrease the net GHG emissions over a certain period of time [29]. Biogenic carbon stored in construction elements should also be considered, as long-term carbon storage can compensate for the loss of carbon in forest ecosystems (Rüter et al. 40). This study focuses on the substitution impacts on the product level, thus excluding biogenic carbon- and market dynamics.

Assessing the substitution impacts of wood construction is a complex undertaking, and it becomes the more uncertain the longer the timeframe. In this study, we focus on two main factors likely to greatly influence the substitution impacts of wood use: the decarbonization of the energy sector and the increase in recycling. The EU’s goal is to cut GHG emissions of energy production by 80–95% by 2050 (European Commission 41). Thus, GHG emissions caused by the manufacture of construction materials may be substantially smaller in the future. As it is assumed that the GHG emissions of construction based on both, wood as well as concrete and steel, will decrease, the climate change mitigation impact through wood construction is becoming more uncertain. As steel and concrete tend to have greater embodied emissions than wood-based construction products, the relative emission reductions can also be greater than for wood-based products, which would diminish the relative benefit of wood use (e.g., [7]). Besides the reduction in energy production emissions, there could be further reductions in the energy intensity of production processes, as well as in the processing related emissions of concrete and steel, such as calcination in the cement production process (e.g., [14]).

Another fundamental factor influencing the mitigation impact of wood construction is the recycling of construction materials. The EU action plan for the circular economy [2] advocates maintaining the value of products, materials, and resources, and to minimize waste generation. The EU Waste Framework Directive (2008/98/EC) stipulates that 70% of non-hazardous construction and demolition waste must be prepared for re-use, be recycled, or undergo other material recovery by 2020. At the time of the Directive’s introduction, the recycling rate of construction waste in the EU27 was on average 63%, and for wood 30%, with significant differences between member states. The recycling of construction materials, especially mineral-based construction materials, is anticipated to increase in the future [1]. This increase will lead to a decrease in emissions of mineral-based construction materials and simultaneously to a decrease of the GHG emissions of buildings. There are also possibilities to recycle wooden construction materials (e.g., using discarded wood as a raw material for particle board composites, etc.), but in many cases they are not economically feasible (e.g., [28]). Technical limitations, as well as the lack of supporting policies, may hamper the recycling of wooden construction products. Consequently, wood construction materials are used for energy after demolition in countries where no economically feasible alternatives for discarded wood exist.

Although wood construction substitution impacts are extensively assessed in the scientific literature, there are few systematic efforts to account for decarbonization and increased recycling. In this study, we assess the substitution impacts of selected wood-frame multi-story building types representative of the Nordic markets with simulations up to 2050, considering the impacts of increased recycling and reduced energy emissions.

More specifically, the aims of this study are:

- To quantify DFs for a fossil GHG reduction when building with wood instead of steel and concrete, using life cycle inventories and impact assessment on selected house types with the same functionality.
- To assess how the decarbonization of the energy sector and increased recycling influences DFs in the future, considering alternative decarbonization scenarios and recycling targets.

## Materials and methods

### System boundaries

**Fig. 1:**
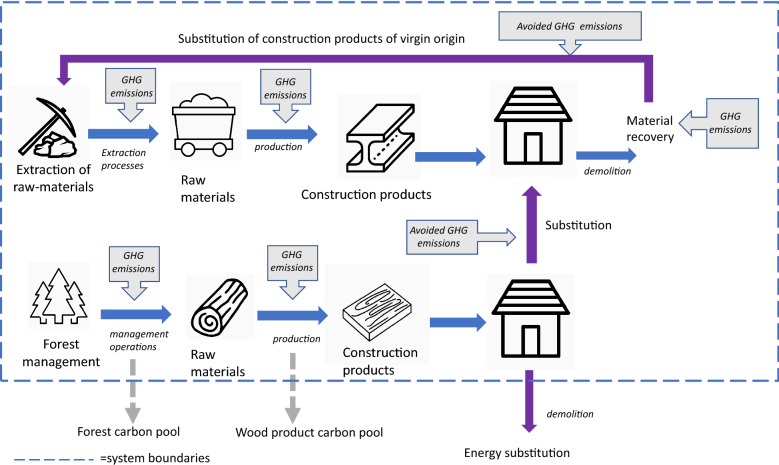
Illustrative flowchart of processes included in the scope of this study

In this study, the substitution impact was assessed by estimating DFs for comparable house types under future scenarios assuming reduced energy sector emissions and increased recycling of construction products. Given the relatively long timeframe required for meeting such targets, the temporal scope of the study was 2020–2050. As the future scenarios contain inevitable uncertainties, we used stochastic simulation approach to determine the DFs by including a range of possible scenarios related to decarbonization of the energy sector and recycling rates of discarded construction products. This assessment is limited to product level DFs, and therefore excludes biogenic carbon emissions and removals as well as market dynamics (Fig. 1).

The impacts of the maintenance and demolition of buildings were not considered. Also, it was assumed that the maintenance and energy use of the alternative house types were identical, as the compared buildings have the same functionality. Using discarded wood as energy can replace fossil energy and provide additional substitution impacts (e.g., [27]). However, determining the emissions of discarded wood from material recycling instead of energy is a complex undertaking. The use of discarded wood for energy can be less favorable in terms of GHG emissions reduction than recycling [5]. Importantly, for wood buildings built today, the possible end-of-life substitution impacts would only be gained well past 2050, by which point the energy sector is expected to have radically reduced its average emissions, leading to smaller relevancy of end-of-life energy recovery of wood products. Therefore, plausible end-of-life substitution credits were left outside the system boundaries.

### Alternative house types

The DFs for construction were assessed using scientific articles and reports from the Nordic countries with similar climate conditions and construction regulations. Three studies report sufficient data on the life cycle inventories and impact assessment of functionally equivalent house types: Tettey et al. [30], Ruuska and Häkkinen et al. [25] and Peñaloza et al. [23]. As it was assumed that the use-phase of house types was similar in terms of energy use and maintenance, only the GHG emissions of manufacturing construction materials were considered.

Tettey et al. [30] assessed a six-storey building with a frame of prefabricated concrete, prefabricated modular timber or cross laminated timber (CLT) elements, designed to meet the Swedish passive house criteria. Ruuska and Häkkinen [25] evaluated a five-storey residential model building with a concrete, CLT or timber frame. The volume of the building was 6108 m^3^, with a respective floor area of 1813 m^2^. Additional file 1 was requested from the authors of the original research paper to complete the calculations for all construction components such as the external walls, roofs, and base floors (see Additional file 1: Tables S1–S3). These components were then calibrated to match with the total mass and volume of the buildings. A study by Peñaloza et al. [23] included data on multi-family houses: three types of timber-based multi-family dwellings (prefabricated volume elements, massive elements, and column-beam); and three types of multi-family dwellings with a concrete structure.

Wood-frame multi-story construction is still in its infancy, with the market share remaining at a few percentage even in the Nordic regions where it has been promoted for decades [32]. So far, the house types described by Tettey et al. [30] and Ruuska and Häkkinen [25] appear to be the most typical structures or are considered to increase significantly in the future [9]. However, the exact market shares are unknown and they remain subject to speculation for future scenarios.

### GHG emissions of construction products, and impacts of future decarbonization and recycling scenarios

The GHG emissions of houses were aggregated using data on material use and GHG emissions of construction products. The GHG emissions of construction products were projected for the period 2020–2050 and were aggregated for each year by considering the decarbonization of the energy sector as well as the impacts of recycling construction products. Data on the GHG emissions of the production of construction products were acquired from various sources, such as the ecoinvent database and GHG emissions reports (see more details in Additional file 1: Table S4).

A set of “what if” scenarios were assumed to estimate the GHG emissions of construction materials in the future. For the sake of simplicity, the GHG emissions of energy production were assumed to decrease in a linear trend between 2020 and 2050. The range of emission reductions varied from 40 to 80% from the level in 2020. The shares of energy emissions for various construction materials were obtained from the ecoinvent 3.0 database (see Additional file 1: Table S7). The calcination of cement was included by assuming that emissions of concrete would decrease 45–75% in 2050 from the level in 2020 [18, 37]. A linear decrease in annual calcination emission reduction was assumed, with an uncertainty range.

There are no binding targets on the recycling rates of different construction materials. As such data were not available, alternative approaches were applied to assess the recycling potential of construction materials in the future scenarios. Data on the recycling rates of construction products were taken from Finnish reports on recycling targets for various materials (see Additional file 1: Table S8).

The process of recycling construction materials, such as transportation to the recycling center and the crushing of concrete, requires some energy input. Consequently, recycling causes GHG emissions, although the amount is typically less than that caused by using virgin raw materials. Data on GHG emissions of recycling construction materials of non-wood origin were taken from Turner et al. [33]. The energy emissions of recycling are also assumed to decrease in the future because of the decarbonization of the energy sector.

The GHG emissions of construction products were aggregated according to Eq. 1:

1 $$GHG_{it} = \left( {1 - REC_{it} } \right)*\left( {1 - EN_{i2020} } \right)*GHG_{i2020} + \left( {1 - REC_{it} } \right)*\left( {EN_{i2020} *ENER_{i} *GHG_{i2020} } \right) + REC_{it} *ENER_{i} *REM_{i2020}$$

where, $REC_{it}$ = *Share of recycled products of material i in year t*, $EN_{i2020}$= *Share of energy emissions of material i in 2020*, $GHG_{i2020}$= GHG emissions *product i in 2020*, $ENER_{i}$= *Share of energy emissions in year t compared to emissions in 2020*, $REM_{i2020}$=*GHG emissions of recycling of material i in 2020*. The emissions were aggregated as kg CO_2_ eg/kg of a construction material.

### Calculation of displacement factors

DFs were calculated for each house type from 2020 to 2050 according to Eq. 2, as provided in Sathre and O’Connor [27]

2 $$DF = \frac{GHG\, nonwood - GHG \, wood}{{WUwood - WUnonwood}}$$

where GHGnonwood and GHGwood include aggregated GHG emissions of the required construction materials, and WUwood and WUnonwood include wood use as biogenic carbon contained in the respective house types. GHGnon-wood and GHGwood were aggregated by multiplying the required raw materials with the carbon footprint of each raw material. WUwood and WUnonwood were calculated assuming that the carbon content of wood-based raw materials is 50%. As buildings based on mineral materials in most cases also include some wood, WUnonwood is typically not zero, although the values are much smaller than in wood-framed buildings (see Additional file 1: Tables S1–S3). However, the DFs are calculated only for substitution cases where a wood-based design replaces a non-wood-based design, because substitution between wood-based products ought to only be considered in the process of upscaling the substitution impacts from a product level to a market level through weighted DFs for intermediate wood-based products [13]. Because of the decarbonization of the energy sector and recycling of construction raw materials, GHGnonwood and GHGwood are assumed to decrease whereas WUwood and WUnonwood remain at the same level. The DFs applied in this study imply that there are no emission leakages, i.e., a unit emission reduction at the building level equals a unit emission reduction to the atmosphere.

S*imulacion* 4.0. Excel add in was used to simulate the DFs in 10-year intervals from 2020 to 2050 for three scenarios: the first with decarbonization of the energy sector and the recycling of discarded construction products; the second including only decarbonization of the energy sector; and the third including only recycling. 1000 simulations were carried out for each scenario. Because of decarbonization and recycling GHG emissions of the construction raw materials are expected to decrease in the future. Furthermore, GHG emissions of construction materials in this study are based on different data sources (see Additional file 1: Tables S5–S8). By including several comparable data sources, we ensured that the variation in GHG emissions of construction products is included as one source of uncertainty along with decarbonization of the energy sector and recycling. For all three variables a uniform distribution was assumed.

### Sensitivity analyses

As concrete, steel and wood are abundantly used in construction, the GHG emissions of these raw materials are assumed to have a substantial impact on the total GHG emissions of a building and on the substitution impacts of wood construction. To assess the sensitivity of the results on the GHG emissions of the presumed most influential raw materials, three alternative scenarios were implemented.

In ‘zero fossil GHG emissions wood’ scenario, it was assumed that the emissions of all wood-based products would be zero in 2050 [3]. This could be achieved by using an even higher rate of bio-based residues or alternative renewable energy sources to cover the operational energy demand of mills. In this scenario, the reduction in emissions is assumed to be caused by increasing the circular use of wood products and by decarbonization of the energy sector. In this setup, the carbon footprints of all wood-based raw materials were zero in 2050. For the sake of simplicity, a linear decrease in emissions from 2020 to 2050 was assumed. In *‘*zero GHG fossil emissions concrete’ scenario, it was assumed that the cement production will reach carbon neutrality by 2050, as envisioned by Cembureau [6]. The target could be reached through alternative clinkers, increased efficiency of production, the use of alternative fuels, better transport efficiency, and breakthrough technologies, such as carbon capture. As cement is the main component causing GHG emissions in concrete production, it was assumed that because of the carbon neutrality of cement, in 2050 the carbon footprint of concrete will be zero. In *‘*zero fossil GHG emissions steel’ scenario, it was assumed that the steel production will reach carbon neutrality by 2050 by using, e.g., top gas recycling blast furnaces, carbon capture and storage, and the substitution of pulverized coal injection with biomass [31]. For all three zero fossil GHG emission scenarios DFs were aggregated using the same approach (see Section “Calculation of displacement factors”).

## Results

### Displacement factors in future scenarios

The DFs calculated for alternative house types were highly variable (Table 1). The majority of DFs were positive, implying decreasing fossil GHG emissions when a wooden house is built instead of an alternative house based on a greater use of concrete and steel. The highest mean DF with 2020 values was for a modular wooden frame versus a concrete frame based on data from Tettey et al. [30]: 2.27 tons of fossil carbon avoided per ton of biogenic carbon contained in wood products. However, four slightly negative average DFs were also detected, indicating increasing GHG emissions, if wood is favored.

**Table 1:** Displacement factors (tC/tC) for various house types

Compared house types	2020			2050			Including decarbonization only			Including recycling only		
				Including decarbonization and recycling								
	Mean	Min	Max	Mean	Min	Max	Mean	Min	Max	Mean	Min	Max
CLT vs concrete^a^	0.98	0.82	1.12	0.19	0.09	0.30	0.41	0.22	0.65	0.40	0.34	0.46
Modular vs concrete^a^	2.27	1.94	2.64	0.46	0.22	0.71	0.97	0.53	1.45	0.96	0.82	1.09
CLT vs concrete^b^	0.09	− 0.04	0.22	− 0.04	− 0.10	0.01	− 0.01	− 0.12	0.10	− 0.10	− 0.16	− 0.02
Timber vs concrete	0.34	− 0.04	0.73	− 0.01	− 0.11	0.08	0.06	− 0.27	0.31	− 0.12	− 0.21	− 0.02
Massive timber elements vs in situ casting^c^	0.63	0.28	1.00	0.09	0.02	0.17	0.23	0.00	0.49	0.14	0.07	0.22
Prefab. elem. vs in situ cast^c^	0.32	− 0.25	0.92	− 0.06	− 0.14	0.03	0.03	− 0.38	0.41	0.03	− 0.38	0.41
Massive elements vs PFH^c^	− 0.01	− 0.50	0.42	− 0.15	− 0.29	− 0.07	− 0.12	− 0.48	0.16	− 0.28	− 0.33	− 0.23
Massive elements vs VST^c^	0.24	0.03	0.47	− 0.03	− 0.09	0.00	0.13	− 0.01	0.27	− 0.08	− 0.11	− 0.04
Column-beam vs in situ casting^c^	− 0.01	− 0.50	0.42	− 0.15	− 0.29	− 0.07	− 0.12	− 0.48	0.16	− 0.28	− 0.33	− 0.23
Prefab. elem vs PFH^c^	0.07	− 0.20	0.35	− 0.05	− 0.11	− 0.02	− 0.01	− 0.27	0.14	− 0.09	− 0.13	− 0.05
Column-beam vs PVF^c^	− 0.48	− 0.83	− 0.12	− 0.20	− 0.27	− 0.12	− 0.25	− 0.52	− 0.05	− 0.41	− 0.43	− 0.40
Column-beam vs vst system^c^	1.28	0.96	1.60	0.12	0.09	0.15	0.74	0.41	1.15	0.17	0.14	0.19
Prefab elem. vs vst system^c^	− 0.09	− 0.50	0.29	− 0.16	− 0.25	− 0.07	− 0.05	− 0.29	0.20	− 0.41	− 0.43	− 0.38

^a^Tettey et al. [30]

^b^Ruuska and Häkkinen 2012

^c^Peñaloza et al. 2018

All DFs, regardless of house type, showed a decreasing trend towards 2050. That is, the fossil GHG mitigation impact of wood construction will decrease in the future, as the emissions of the respective construction practices decline more relative to the emissions of wood-based products. In the simulation rounds where both the decarbonization of the energy sector and recycling of construction materials were considered, the DFs substantially decreased from 2020 to 2050.For example, the substitution case with the highest DF (modular vs. concrete) decreased from 2.27 in 2020 to 0.46 tC/tC in 2050. In the simulations where only the decarbonization of the energy sector was included, the decrease was less dramatic compared to the isolated impact of recycling construction materials. In some cases, the decrease in DFs was smaller when only recycling was considered than in cases where both the decarbonization of the energy sector and recycling were considered. However, in some simulations an opposite result was drawn.

### Impact of zero fossil GHG emission scenarios

**Fig. 2:**
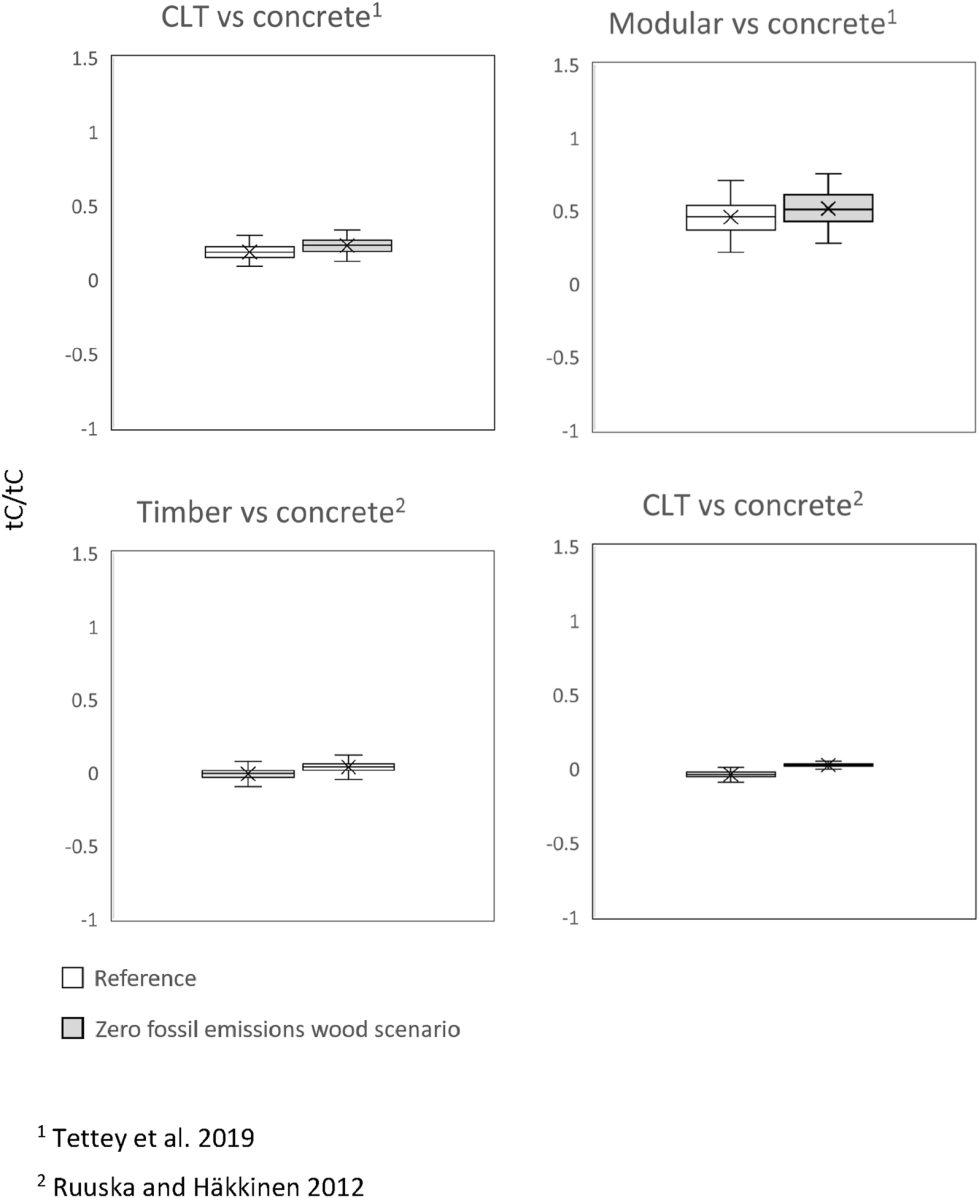
DFs in 2050 for the *zero fossil GHG emissions wood scenario*. The central line indicates the median, the bottom and top edges of the box are the 25th and 75th percentiles and the whisker indicates the minimum and maximum values (outliers excluded)

The simulation of DFs with zero fossil GHG emissions for wood in 2050 were compared to those where a more subtle decrease in emissions was assumed. In the *zero fossil GHG emissions wood scenario*, the DFs based on Tettey et al. [30] (Fig. 2) were similar to the scenario where a more subtle decrease in GHG emissions was assumed. It appears that even if the wood construction raw materials were fossil emission free in the future, there would be no significant changes in the DFs,.

**Fig. 3:**
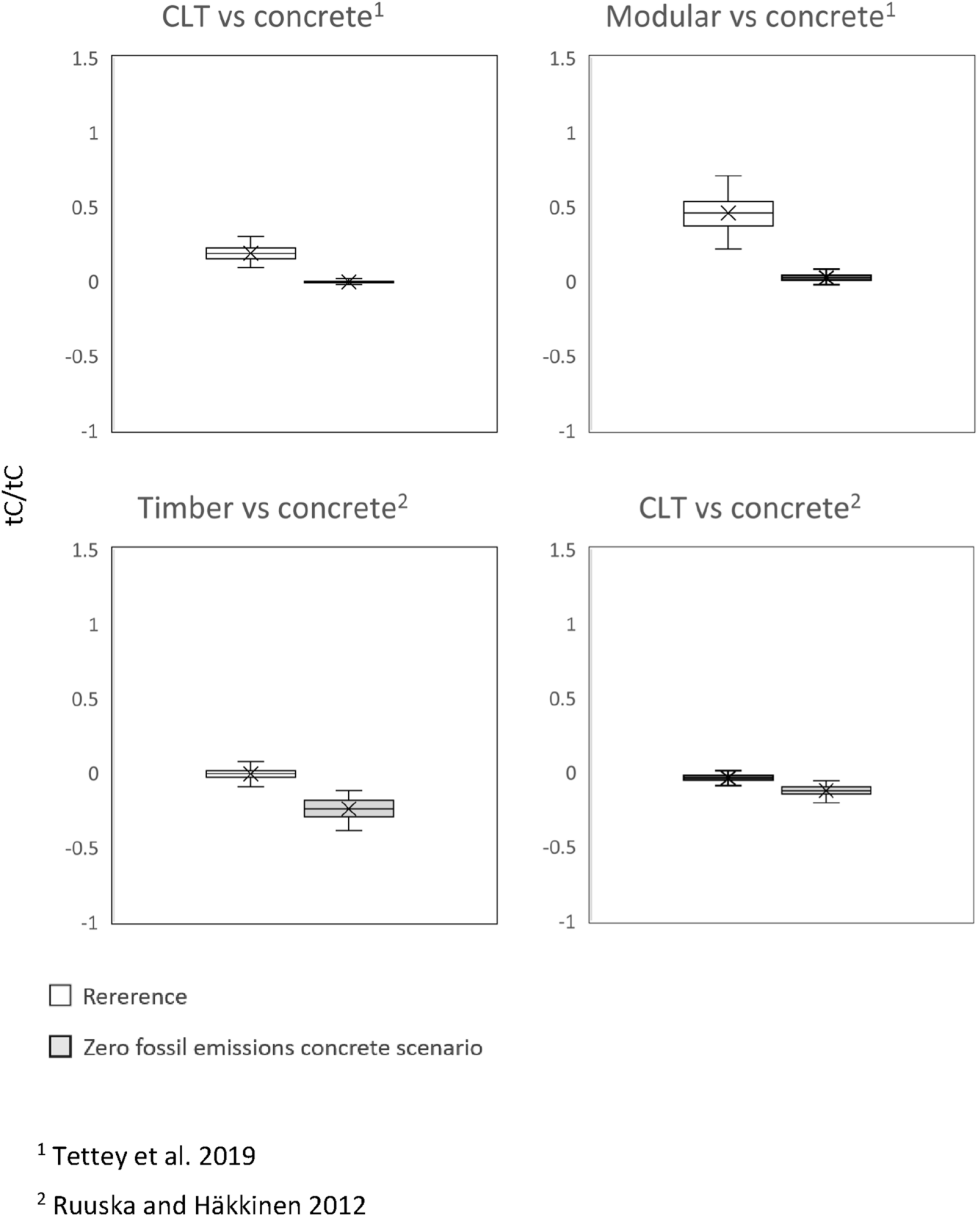
DFs in 2050 for the *zero fossil GHG emissions concrete scenario*. The central line indicates the median, the bottom and top edges of the box are the 25th and 75th percentiles and the whisker indicates the minimum and maximum values (outliers excluded)

In the *zero fossil emissions concrete scenario* (Fig. 3), all DFs were smaller than in the scenario where a less dramatic decrease in the GHG emissions of concrete was assumed. In other words, if concrete production were emission free in the future, this would significantly change the DFs of wood construction.

**Fig. 4:**
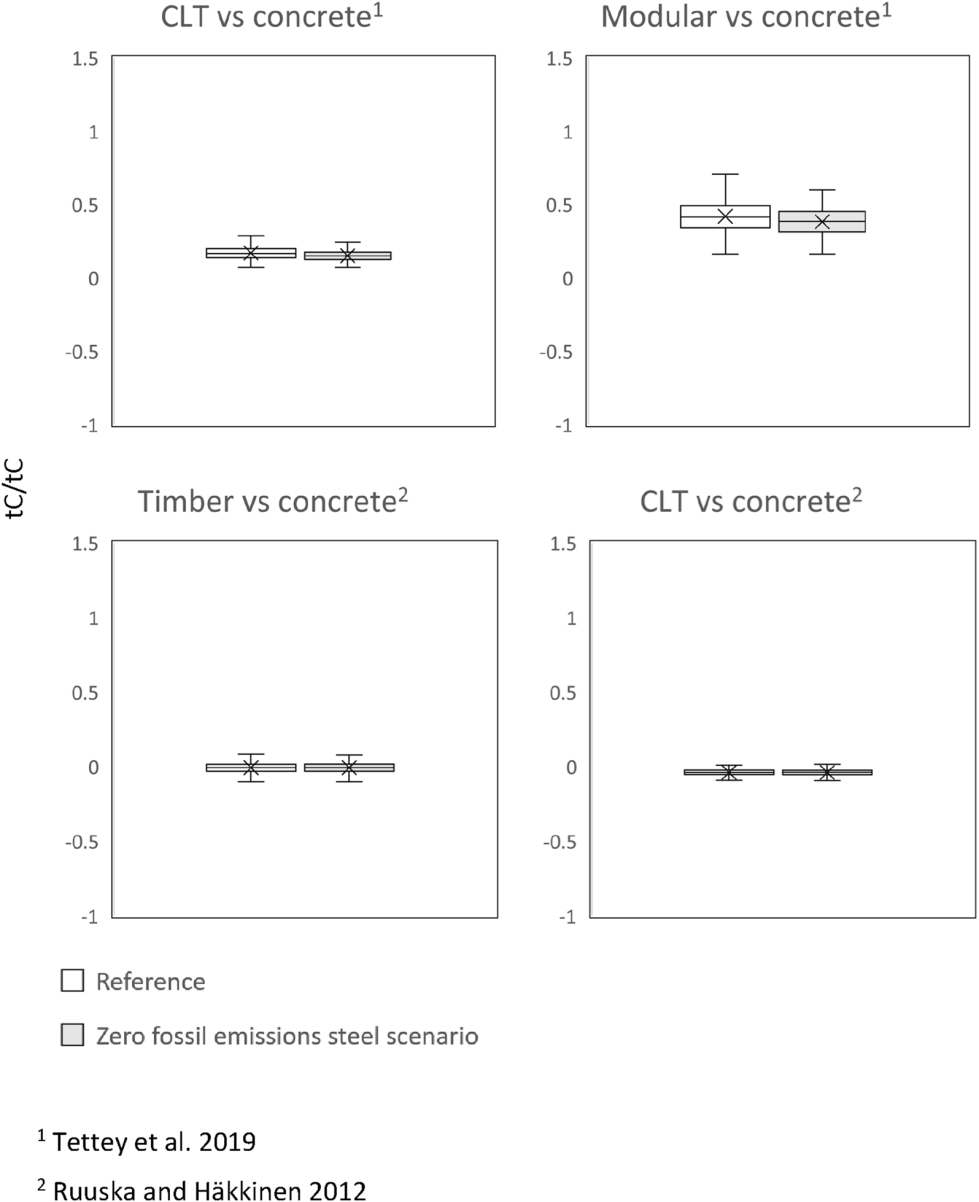
DFs in 2050 for the *zero fossil GHG emissions steel scenario*. The central line indicates the median, the bottom and top edges of the box are the 25th and 75th percentiles and the whisker indicates the minimum and maximum values (outliers excluded)

In the *zero fossil emissions steel scenario* (Fig. 4), the DFs were different from the reference DFs, but there was no clear trend in the change.

## Discussion

### A decreasing trend for substitution impacts of wood use

In this study, we assessed the relative impact of the decrease in the fossil GHG emissions of the energy sector on the substitution impacts of wood construction by estimating displacement factors (DFs) for alternative house types through scenario analysis and uncertainty assessment. Using current data, the DFs were positive in most cases, implying that the use of wood reduces fossil GHG emissions.

In this study, the DFs for construction were highly variable. Previous studies also address the huge variation in DFs. Geng et al. [16] demonstrated that the replacement of reinforced concrete nonresidential buildings in China with wood construction can provide the greatest DF (6.81 tC/tC), while a brick-concrete residential building (bungalow) provided the smallest DF (1.51 tC/tC). In their meta-analysis, Sathre and O’Connor [27] estimated a range of DFs for construction, from a low of − 2.3 to a high of 15 tC/tC, with most DFs lying in the range of 1.0–3.0 tC/tC. In their study, however, the case studies are based on different system boundaries and are not directly comparable to the DFs in this study. Given the high variation, using a single average DF for all construction products may oversimplify the analysis of substitution impacts and thus produce large errors in the estimates.

Comparisons of DFs between the different building designs are not necessarily fruitful, as the individual DFs vary significantly from one house type to the next, and within a single house type, depending on the data source. This is probably at least partly explained by case-by-case building designs, in which the amount of wood used versus the amount of concrete and steel used can vary significantly, even within a specific type of building structure. Due to this variation, the level of the DFs cannot be generalized beyond these individual cases. Thus, the main significance of the results are the impacts of the three scenarios compared to the baseline within each substitution case. To partly address the possible impact of the variation in building design on the scenario results, a series of sensitivity analyses were conducted.

According to the sensitivity analyses, the emissions of wood construction materials themselves are far less influential for the substitution impacts than the emissions of concrete. In this study, it appeared that even if a zero fossil emissions wood scenario was to be actualized, this would not have a significant impact on the DFs. Also, Geng et al. [16] concluded that when assessing the substitution impacts of wood construction, the emissions of non-wood construction products are more influential than the emissions of wood materials. This is likely a result of the low fossil fuel input in the manufacture of wood-based products to begin with. That is, the sidestreams of sawmilling are utilized in part to supply the energy for the manufacture of sawnwood, and biogenic emissions from biomass combustion are calculated as zero in the energy sector to avoid double counting with the biogenic carbon stock changes in the land use, land use change and forestry (LULUCF) sector in national GHG inventories (e.g., [24]).

Another influential factor in decreasing the emission reduction impact of wood is the increased rate of recycling. In this study, the GHG emissions of recycled construction materials were found to be much smaller than those of materials based on virgin raw materials; thus, houses with a large share of recycled raw materials cause lower GHG emissions.

Although it was assumed that wood-based construction materials would not be recycled, the GHG emissions of wood-based buildings decreased in the scenarios in which only recycling was included. This is because the GHG emissions of other construction materials used in wood-based construction, such as insulation and plastics, efficiently decreased their emissions because of recycling. Thus, recycling has an adverse impact on the DFs. This study did not consider the possible increase in the recycling of wood products (often termed cascading), as it has not been considered to hold significant potential in the Nordic contexts (e.g., [10].

This study included several cases of functionally equivalent wood- and alternative buildings based on concrete and steel as the primary building materials. As large-scale wood construction systems are still far from being standardized, the building types studied may not be the principal house types built in the future. The construction sector is seeking new construction techniques and innovative raw materials not assessed in this study. Therefore, it is possible that in the future there will be new alternative wood house types available with a different substitution impact than the houses assessed in this study, for example, utilizing nanocellulose structures [11].

### From product level to market level substitution impacts

Our analysis was executed at the building level, comparing single functional units to one another. This is merely an intermediate step, yet crucial, in assessing the climate impacts of wood construction over time. An expansion of system boundaries and several additional assumptions are required for upscaling the substitution impacts from single cases to the entire wood construction market.

Importantly, this study provides insights only into the fossil GHG emissions balance, not a full account of the climate impacts of wood construction. To study the latter, one must define market scenarios for marginal changes in wood use and define the consequences not only for the overall substitution impacts, but also the biogenic carbon emissions and removals in forest ecosystems and wood products (e.g., [17]). Moreover, this net impact needs to be assessed in different timeframes, due to the dynamic nature of biogenic emissions and removals (e.g., [35]).

While accounting for the decarbonization and increased recycling facilitates drawing more accurate substitution impact estimates, several challenges remain for the upscaling of substitution impacts from product to market level. Notably, it is often unclear, which wood-based product can be assumed to replace which non-wood product, and to what extent [8], even though this could be less of an issue for construction products than for many other wood-based products, due to clearly defined functional equivalence. Related to this, wood-based products or wood-based building designs are also likely substituting one another, which is typically ignored in market level substitution impact estimates. Substitution between wood-based products themselves can be assessed by using DFs, but only indirectly through weighting different end uses of intermediate products, as the DFs only apply for wood products substituting non-wood products.

Ideally, one ought to additionally consider price-mediated market responses to changes in wood uses, such as leakages and rebound effects. Besides international carbon leakage, there is likely to be intersectoral or intertemporal carbon leakage [7]. That is, one additional unit of fossil emissions avoided in the construction sector may not necessarily lead to a unit reduction of fossil emissions to the atmosphere. If wood replaces concrete in the construction sector, the avoided use of fossil feedstocks may end up being used in another sector, or substitution may simply delay the use of fossil feedstocks [7]. Moreover, secondary consequences may either hinder substitution or multiply consequent impacts. For example, a price rise associated with increased biomass use can increase the demand for fossil-based products (rebound effect) or the upstream supplier impacts can exceed the direct impacts (multiplier effect) [12].

One cannot draw direct policy implications from case specific DFs, as these merely represent an intermediate step in the broader analysis of the climate impacts of the forest sector and the GHG dynamics between the LULUCF sector, the energy sector, and industrial sectors. In order to assess the total GHG impacts of wood use, it is imperative to assess the overall product portfolio of industries, including all primary products, the use of by-products, and the energy uses of wood. More than 50% of the sawlogs used for wood construction end up in energy production or pulp manufacturing in the form of sawdust and chips, which have, on average, lower substitution impacts compared to wood construction. Given the current structure of the industry, the overall substitution impacts are likely to be lower for total wood use than for the construction sector.

The results showed that the decarbonization of the energy sector and recycling of construction products are highly influential factors when assessing the substitution impacts of wood construction. When both decarbonization of the energy sector and recycling are considered, there is a dramatic decrease in DFs in the future. Even if the emissions of wood-based products were to diminish, the reduction in the emissions of steel and concrete would still be relatively higher, resulting in declining DFs. The results support the hypotheses that the decarbonization of the energy sector and recycling of construction products will substantially lower the DFs for wood products. This will lead to a highly significant overall reduction of emissions in the construction sector. In such a scenario, overall lifetime of a building and carbon storage aspects in wood construction might be more relevant policy decision points than material substitution.

## Conclusions

This study substantiates the findings of a large range of studies which indicate that wood construction causes less fossil GHG emissions in the technosystem than a comparable house based on concrete. However, the scale of this impact is highly varied and depends on the building designs.

Although the DFs for different house types were highly variable, nearly all DFs were estimated to decrease in the future. The overall aim, however, should not be to maximize DFs but to decrease overall emissions. Tools, means, and policies to enhance e.g., recycling and resource efficiency often imply smaller emissions for both wood and non-wood products, but the relatively bigger emission reduction in the non-wood technologies leads to smaller substitution impacts for wood. Resource-efficiency and minimizing material waste should also become a policy target alongside climate change mitigation. Thus, the described scenarios indicate a positive trend in terms of mitigating climate change, due to overall lower GHG emissions. For the wood products sector, however, society with lower GHG emissions would mean entering a new competitive situation, as there would be fewer emissions to be avoided through substitution with wood. It is crucial to also acknowledge that with wood construction it is not possible to substitute all buildings based on mineral raw materials, as construction always involves the use of a mix of materials and because wood is a limited, though renewable resource in the global context. Therefore, it is highly important to minimize the emissions of mineral raw materials as substitution represents only one option to reduce the emissions of the construction sector.

The decarbonization of the energy sector and recycling of construction products are highly influential factors when assessing the substitution impacts of wood construction. When both decarbonization of the energy sector and recycling are considered, there is a dramatic decrease in the DFs. Thus, a reassessment of DFs needs to be undertaken, as the decarbonization and recycling rates begin to significantly increase and as data becomes available. This is a highly positive trend but it emphasizes the need to reassess the substitution impacts of wood construction. When the substitution impacts are reduced, the carbon storage in wood products will become relatively more relevant.

This study strongly suggests that the substitution impacts of wood construction are highly sensitive to changes in the technosystem when it comes to the decarbonization of the energy sector and recycling of discarded construction products. Ambitious targets exist to decarbonize the energy sector and strengthen the circular use of materials. If these targets are reached, the overall GHG emissions of the construction sector will be significantly reduced.

## Supplementary Information

**Additional file 1: Table S1.** Materials required (as tonnes) of major materials in the finished building alternatives from Tettey et al. [30]. **Table S2.** Materials required (kg/building) for alternative buildings obtained from Vares et al. (2017). **Table S3.** Materials required for alternative buildings (kg) per heated floor area obtained from Peñaloza et al. (2018). **Table S4.** Carbon footprints of wooden construction materials (kg CO2 eq./kg). **Table S5.** Carbon footprints of plastic construction materials (kg CO_2_ eq./kg). **Table S6.** Carbon footprints of other construction materials (kg CO2eq/kg). **Table S7.** Share of GHG energy emissions caused of the total carbon footprint of a construction material (Ecoinvent 3.0). **Table S8.** Assumptions behind recycling scenarios.

## Abbreviations

CLT: Cross laminated timber
DF: Displacement factor
GHG: Greenhouse gas
LULUCF: Land use, land use change and forestry
