# Supplementary material for: Substitution impacts of Nordic wood-based multi-story building types: influence of the decarbonization of the energy sector and increased recycling of construction materials
Source: Carbon Balance Manag. 2022 May 17;17:4. doi: 10.1186/s13021-022-00205-x (PMC9115976; doi:10.1186/s13021-022-00205-x)
Supplement: Supplementary file 1 — Additional file 1: Table S1. Materials required (as tonnes) of major materials in the finished building alternatives from Tettey et al. [30]. Table S2. Materials required (kg/building) for alternative buildings obtained from Vares et al. (2017). Table S3. Materials required for alternative buildings (kg) per heated floor area obtained from Peñaloza et al. (2018). Table S4. Carbon footprints of wooden construction materials (kg CO2 eq./kg). Table S5. Carbon footprints of plastic construction materials (kg CO2 eq./kg). Table S6. Carbon footprints of other construction materials (kg CO2eq/kg). Table S7. Share of GHG energy emissions caused of the total carbon footprint of a construction material (Ecoinvent 3.0). Table S8. Assumptions behind recycling scenarios. [file 13021_2022_205_MOESM1_ESM.docx]

**Substitution impacts of wood construction: The influence of decarbonization of the energy sector and circular use of construction materials**

|  |  | **Concrete** | **CLT** | **Modular** |
| --- | --- | --- | --- | --- |
| Concrete |  | 2867800 | 229100 | 229100 |
| Steel |  | 95200 | 12600 | 14200 |
| Wood | | 50900 | 127400 | 153500 |
| Particle board |  | 20800 | 0 | 23 |
| Plywood |  | 3 | 20900 | 29000 |
| CLT (Cross Laminated Timber) |  | 0 | 175700 | 0 |
| Glulam | | 0 | 40 | 8 |
| Rock wool | | 11 | 27 | 6 |
| Glass wool | | 0 | 0 | 19 |
| Expanded Polystyrene | | 14 | 2 | 2 |
| Gypsum board |  | 23 | 110 | 116 |
| sum |  | 3034750 | 565879 | 425974 |

Tanja Myllyviita^a^*, Elias Hurmekoski^b^ , Janni Kunttu^c^

*Corresponding author, tanja.myllyviita@syke.fi, +358 295 251 872

^a^Finnish Environment institute

^b^University of Helsinki

^c^European Forest Institute

Table S1. Materials required (as tonnes) of major materials in the finished building alternatives from Tettey et al. (2019).

Table S2. Materials required (kg/building) for alternative buildings obtained from Vares et al. (2017)

|  | **CLT** | **Concrete** | **Timber** |
| --- | --- | --- | --- |
| CLT (Cross Laminated Timber) | 299788 |  |  |
| Wood | 70532.2 |  | 119927.5 |
| Wood fibre board | 1651.0 |  | 1939.2 |
| Plywood | 2892.1 |  | 14460.4 |
| Polypropene | 389.1 |  | 389.1 |
| Mineral wool | 34747.4 |  | 31386.7 |
| Crushed stone | 91812.0 |  |  |
| Steel | 1963.9 |  | 419.5 |
| Concrete | 15950.0 | 1151732.0 | 15950.0 |
| Plaster | 163950.0 | 5436.8 | 163950.0 |
| Gravel | 147555.0 | 147555.0 | 147555.0 |
| Polystyrene | 327.9 |  | 327.9 |
| Gypsum | 50675.2 |  | 85481.0 |
| Expanded polystyrene | 2377.3 |  |  |
| Polyurethane | | 12956.0 |  |
| Bitumen |  | 5020.0 |  |
| Lightweight aggregate | | 18125 |  |
| Concrete, hollow core slab | | 596876.1 |  |
| Kraft paper | |  | 553.7 |
| Stone wool | |  | 2949.8 |
| Sum | 884611 | 1937701 | 585289.7 |

Table S3. Materials required for alternative buildings (kg) per heated floor area obtained from Peñaloza et al. (2018).

|  | **Timber construction** | | | | **Concrete construction multi-family** | | | **Steel construction** |
| --- | --- | --- | --- | --- | --- | --- | --- | --- |
|  | **Multi-family housing** | | | **Single-family** |  |  |  |  |
|  | **Prefabricated elements** | **Massive elements** | **Column-beam** |  | **In-situ casting** | **VST-system** | **PFH env. optimised** | **Su & Zhang no 1** |
| Concrete (foundation) | 361.6 | 361.6 | 335.9 | 474.5 | 129.1 | 17.0 | 90.3 | 359.2 |
| Low-impact concrete | 0.0 | 0.0 | 0.0 | 0.0 | 0.0 | 0.0 | 223.7 | 0.0 |
| Precast concrete | 0.0 | 0.0 | 0.0 | 0.0 | 244.4 | 69.4 | 23.9 | 225.6 |
| Concrete (normal) | 0.0 | 0.0 | 80.2 | 0.0 | 688.9 | 102.1 | 0.0 | 880.9 |
| Mortar | 9.4 | 14.8 | 9.4 | 0.0 | 0.0 | 0.0 | 18.4 | 0.0 |
| Structural steel | 0.0 | 0.0 | 0.0 | 0.0 | 0.0 | 0.0 | 0.0 | 64.7 |
| Steel (plates, jointing, profiles) | 1.1 | 1.7 | 5.4 | 5.0 | 0.6 | 1.7 | 28.9 | 3.1 |
| Steel (reinforcement) | 1.5 | 1.5 | 3.5 | 0.0 | 30.8 | 13.1 | 23.2 | 28.6 |
| Sawn timber | 52.4 | 40.2 | 21.1 | 18.0 | 9.8 | 1.2 | 0.0 | 0.0 |
| Fiber cement | 0.0 | 0.0 | 0.0 | 3.4 | 0.0 | 0.0 | 0.0 | 0.0 |
| Paper board | 0.0 | 0.0 | 0.0 | 1.4 | 0.0 | 0.0 | 0.0 | 0.0 |
| Particle board | 13.1 | 2.7 | 0.0 | 0.0 | 0.9 | 0.3 | 0.0 | 0.0 |
| LVL (Laminated veneer lumber) | 0.0 | 0.0 | 51.0 | 0.0 | 0.0 | 0.0 | 0.0 | 0.0 |
| Plywood | 7.7 | 5.6 | 0.0 | 6.7 | 0.7 | 0.3 | 0.0 | 0.0 |
| Glulam | 6.9 | 16.9 | 20.9 | 59.3 | 11.8 | 1.3 | 0.0 | 0.0 |
| CLT (Cross Laminated Timber) | 4.1 | 46.1 | 4.1 | 0.0 | 0.0 | 0.0 | 0.0 | 0.0 |
| MDF (Medium-density fibreboard) | 0.0 | 0.0 | 0.0 | 8.0 | 0.0 | 0.0 | 0.0 | 0.0 |
| Glass wool | 13.9 | 0.0 | 5.2 | 41.1 | 0.0 | 0.0 | 0.0 | 0.0 |
| Stone wool | 2.0 | 21.9 | 13.5 | 0.0 | 6.2 | 8.7 | 0.0 | 0.0 |
| Glass and expanded glass | 5.4 | 0.0 | 0.1 | 0.0 | 4.4 | 8.2 | 0.0 | 0.0 |
| Gypsum board | 88.1 | 60.7 | 82.8 | 55.7 | 4.4 | 1.3 | 1.1 | 0.0 |
| Glass-reinforced plastic GRP | 2.6 | 1.6 | 1.2 | 0.0 | 0.0 | 0.0 | 0.0 | 0.0 |
| Expanded Polystyrene | 2.3 | 2.3 | 2.4 | 8.7 | 7.1 | 32.3 | 5.9 | 0.0 |
| Polyethylene (incl. LD and HD) | 0.3 | 0.4 | 0.5 | 4.1 | 1.0 | 2.6 | 0.0 | 0.0 |
| Polypropylene | 0.2 | 0.1 | 0.4 | 0.0 | 1.0 | 2.5 | 0.0 | 0.0 |
| PVC - Polyvinylchloride | 0.2 | 0.2 | 0.2 | 0.0 | 0.0 | 0.0 | 0.0 | 0.0 |
| Polyurethane | 0.1 | 0.4 | 2.3 | 0.0 | 0.0 | 0.0 | 0.0 | 0.0 |
| Synthetic rubber | 0.0 | 0.2 | 0.0 | 0.0 | 0.0 | 0.0 | 0.0 | 0.0 |
| Sum | 572.9 | 578.9 | 640.1 | 685.9 | 1141.1 | 262.0 | 415.3 | 1562.0 |

Table S4. Carbon footprints of wooden construction materials (kg CO2 eq./kg)

| **Particle board** | **Plywood** | **Sawn wood** | **CLT** | **Glulam** | **Wood** | **Wood fibre board** | **Fibre board** | **LVL** | **Source** |
| --- | --- | --- | --- | --- | --- | --- | --- | --- | --- |
| 0.4 | 0.6 | 0.1 | 0.12 | 0.1 |  |  |  |  | Ruuska (2013) |
|  | 0.66 |  | 0.15 |  | 0.1 | 0.35 | 0.425 |  | Ruuska and Häkkinen (2013) |
| 0.61 |  |  | 0.31 |  |  |  |  |  | Häkkinen (2011) |
|  | 0.22 | 0.11 | 0.19 | 0.19 |  |  |  | 0.48 | Peñaloza et al. (2018) |

Table S5. Carbon footprints of plastic construction materials (kg CO_2_ eq./kg)

| **Polyurethane** | **Expanded polystyrene** | **Polypropene** | **Polystyrene** | **PE-plastic** | **Glass-reinforced plastic GRP** | **EPS - Expanded polystyrene** | **Polyethylene (incl. LD and HD)** | **Polypropylene PP** | **PVC - Polyvinylchloride** | **Polyurethane - PUR** | **Synthetic rubber** | **Source** |
| --- | --- | --- | --- | --- | --- | --- | --- | --- | --- | --- | --- | --- |
|  |  |  |  |  |  | 3.3 |  |  |  |  |  | Ruuska (2013) |
| 4.2 | 3.3 | 2 | 3.3 |  |  |  |  |  |  |  |  | Ruuska and Häkkinen (2013) |
|  | 3.41 |  |  | 2.27 |  |  |  |  |  |  |  | Häkkinen (2011) |
|  |  |  |  |  |  | 3.41 |  |  |  |  |  | Ecoinvent database |
|  |  |  |  |  | 2.09 |  | 2.62 | 2.66 | 5.84 | 4.81 | 2.69 | Peñaloza et al. (2018) |

Table S6. Carbon footprints of other construction materials (kg CO2eq/kg).

| **Concrete,hollow core s** | **Concrete** | **Gravel** | **Bitumen** | **Lightweight aggregate** | **Mineral wool** | **Crushed stone** | **Steel** | **Plaster** | **Gypsum** | **Aluminum** | **Glass** | **Glass wool** | **Rock wool** | **Source** |
| --- | --- | --- | --- | --- | --- | --- | --- | --- | --- | --- | --- | --- | --- | --- |
| 0.1 |  |  |  |  |  |  |  |  |  |  |  |  |  | Ruuska (2013) |
| 0.18 | 0.124 | 0.0033 | 0.68 | 0.24 | 0.99 | 0.014 | 1.86 | 0.36 | 1.6 |  |  |  |  | Ruuska and Häkkinen (2013) |
|  | 0.15 | 0 | 0.8 |  | 1.03 |  | 1.1 | 0.36 | 0.42 | 3.25 | 0.54 |  |  | Häkkinen (2011) |
|  | 0.124 |  |  |  |  |  | 1.24 |  |  |  |  | 1.08 | 1.49 | Ecoinvent database |
|  | 0.13 |  |  |  |  |  | 4.71 | 0.82 |  |  |  | 1.27 | 1.17 | Peñaloza et al. (2018) |

Table S7. Share of GHG energy emissions caused of the total carbon footprint of a construction material (Ecoinvent 3.0).

| **Construction material** | **Share of energy emissions** |
| --- | --- |
| Particle board | 0.8 |
| Plywood | 0.68 |
| sawn wood | 0.68 |
| CLT | 0.68 |
| Glulam | 0.72 |
| Concrete, hollow core slab | 0.3 |
| Concrete | 0.3 |
| Polyurethane | 0.78 |
| Expanded polystyrene | 0.78 |
| Gravel | 0.8 |
| Bitumen | 0.8 |
| Lightweight aggregate | 0.8 |
| Wood | 0.8 |
| Wood fibre board | 0.8 |
| Polypropene | 0.78 |
| Mineral wool | 0.8 |
| Crushed stone | 0.8 |
| Steel | 0.78 |
| Plaster | 0.78 |
| Polystyrene | 0.78 |
| Gypsum | 0.78 |
| PE-plastic | 0.78 |
| Glass | 0.64 |
| Glass wool | 0.8 |
| Rock wool | 0.55 |
| Fibre board | 0.9 |
| Laminated veneer lumber (LVL) | 0.68 |
| Glass-reinforced plastic GRP | 0.78 |
| EPS - Expanded polystyrene | 0.78 |
| Polyethylene (incl. LD and HD) | 0.78 |
| Polypropylene PP | 0.78 |
| PVC - Polyvinylchloride | 0.78 |
| Polyurethane - PUR | 0.78 |
| Synthetic rubber | 0.78 |
| Glass and expanded glass | 0.64 |

Table S8. Assumptions behind recycling scenarios

| **Construction material** | **Share of recycled construction materials in 2050** | **Emissions of recycling in 2020 (kg CO_2_ eq/kg)** |
| --- | --- | --- |
| Concrete, hollow core slab | 0.80 | 0.06 |
| Concrete | 0.68 | 0.06 |
| Polyurethane | 0.68 | 0.38 |
| Expanded polystyrene | 0.68 | 0.38 |
| Gravel | 0.80 | 0.04 |
| Lightweight aggregate | 0.00 | 0.10 |
| Polypropene | 0.68 | 0.38 |
| Mineral wool | 1.00 | 0.23 |
| Crushed stone | 0.80 | 0.04 |
| Steel | 1.00 | 0.50 |
| Plaster | 0.68 | 0.66 |
| Polystyrene | 0.68 | 0.38 |
| Gypsum | 1.00 | 0.66 |
| Aluminum | 1.00 | 1.11 |
| PE-plastic | 0.68 | 0.15 |
| Glass | 1.00 | 0.40 |
| Glass wool | 1.00 | 0.85 |
| Rock wool | 1.00 | 0.23 |
| EPS - Expanded polystyrene | 0.68 | 0.15 |
| Polyethylene (incl. LD and HD) | 0.68 | 0.15 |
| Polypropylene PP | 0.68 | 0.15 |
| PVC - Polyvinylchloride | 0.68 | 0.15 |
| Polyurethane - PUR | 0.68 | 0.15 |
| Synthetic rubber | 0.68 | 0.15 |
| Glass & expanded glass | 0.88 | 0.40 |
| Gypsum board | 1.00 | 0.66 |
| Glass-reinforced plastic GRP | 0.68 | 0.15 |
| EPS - Expanded polystyrene | 0.68 | 0.15 |
| Polyethylene (incl. LD and HD) | 0.68 | 0.15 |
| Polypropylene PP | 0.68 | 0.15 |
| PVC - Polyvinylchloride | 0.68 | 0.15 |
| Polyurethane - PUR | 0.68 | 0.15 |
| Synthetic rubber | 0.68 | 0.02 |

**References**

Peñaloza, D., Erlandsson, M., Berlin, J. et al. (2018). Future scenarios for climate mitigation of new construction in Sweden: Effects of different technological pathways. *Journal of Cleaner Production***187**, 2018, 1025–1035.

Tettey, U.Y.A., Dodoo, A., Gustavsson. L. (2019). Effect of different frame materials on the primary energy use of a multi storey residential building in a life cycle perspective. *Energy and Buildings* **185**, 259–271

Vares, S., Häkkinen. T., Vainio, T. (2017). Rakentamisen hiilivarasto. VTT Technical Research Centre of Finland. VTT Asiakasraportti. No. VTT-CR-04958-17 Available at:

<http://www.ym.fi/download/noname/%7B2859F537-ECD2-479D-A62B-F13AD75403F2%7D/136827>

Wernet, G., Bauer, C., Steubing, B., Reinhard, J., Moreno-Ruiz, E., and Weidema, B., 2016. The ecoinvent database version 3 (part I): overview and methodology. The International Journal of Life Cycle Assessment, [online] 21(9), pp.1218–1230.
